# Etymologia: Quarantine

**DOI:** 10.3201/eid1902.ET1902

**Published:** 2013-02

**Keywords:** etymologia, quarantine, isolation, plague, Black Death, 40, Dubrovnik

**Quarantine [kwor′ən-tēn]**

From the Italian *quaranta* (forty), “quarantine” refers to the practice established in European port cities during the Black Death requiring vessels to lie at anchor for 40 days before landing. Isolation (from the Latin *insula* or island), the practice of separating sick persons from those who are healthy to prevent spread of disease, goes back a long time. “As long as they have the disease they remain unclean. They must live alone; they must live outside the camp” (Leviticus 13:46).

Quarantine, on the other hand, is the practice of separating persons who appear to be healthy but may have been exposed to a disease. In 1377, the Great Council of Ragusa (modern-day Dubrovnik) established a 30-day separation period (*trentino*) for visitors from plague-endemic areas. In the following decades, the practice spread to other cities and was extended from 30 to 40 days (*quarantino*). The longer period may have been more effective at preventing disease or just a nod to the 40-day duration of Biblical events―the Great Flood or Jesus’ fast in the wilderness.
